# Red Beetroot Extract Abrogates Chlorpyrifos-Induced Cortical Damage in Rats

**DOI:** 10.1155/2020/2963020

**Published:** 2020-03-09

**Authors:** Gadah Albasher, Asma S. Alsaleh, Nourah Alkubaisi, Saleh Alfarraj, Saad Alkahtani, Manal Farhood, Nouf Alotibi, Rafa Almeer

**Affiliations:** ^1^King Saud University, Department of Zoology, College of Science, Saudi Arabia; ^2^King Saud University, Department of Botany and Microbiology, College of Science, Saudi Arabia; ^3^King Saud University, Department of Biochemistry, College of Science, Saudi Arabia

## Abstract

Organophosphorus insecticides including chlorpyrifos (CPF) are mainly used for agriculture, household, and military purposes; their application is associated with various adverse reactions in animals and humans. This study was conducted to evaluate the potential neuroprotective effect of red beetroot methanolic extract (RBR) against CPF-induced cortical damage. Twenty-eight adult male Wistar albino rats were divided into 4 groups (*n* = 7 in each group): the control group was administered physiological saline (0.9% NaCl), the CPF group was administered CPF (10 mg/kg), the RBR group was administered RBR (300 mg/kg), and the RBR+CPF group was treated with RBR (300 mg/kg) 1 hr before CPF (10 mg/kg) supplementation. All groups were treated for 28 days. Rats exposed to CPF exhibited a significant decrease in cortical acetylcholinesterase activity and brain-derived neurotrophic factor and a decrease in glial fibrillary acidic protein. CPF intoxication increased lipid peroxidation, inducible nitric oxide synthase expression, and nitric oxide production. This was accompanied by a decrease in glutathione content and in the activities of glutathione peroxidase, glutathione reductase, superoxide dismutase, and catalase in the cortical tissue. Additionally, CPF enhanced inflammatory response, indicated by increased levels and expression of interleukin-1*β* and tumor necrosis factor-*α*. CPF triggered neuronal apoptosis by upregulating Bax and caspase-3 and downregulating Bcl-2. However, RBR reversed the induced neuronal alterations following CPF intoxication. Our findings suggest that RBR can minimize and prevent CPF neurotoxicity through its antioxidant, anti-inflammatory, and antiapoptotic activities.

## 1. Introduction

Chlorpyrifos (CPF) is a wide-spectrum organophosphorus pesticide used for the cultivation of different crops worldwide [1]. CPF intoxication results from intentional, occupational, and accidental exposure, inducing severe adverse reactions in different body organs including the liver, kidney, testes, and central nervous system [2, 3]. It has been reported that CPF can cross the blood-brain barrier [4]. Exposure to CPF even at low doses has been associated with several deleterious neurological impairments such as disturbed blood-brain barrier integrity, attention deficit hyperactivity disorder, Parkinsonism, and dementia in human and animal studies [4, 5]. The inhibition of acetylcholinesterase (AChE) is the primary mechanism resulting in disturbances in cholinergic transmission [6]. Neuroinflammation, apoptosis, and molecular alterations in the nervous tissue following CPF exposure have also been reported as potential underlying mechanisms [6]. CPF induces excess reactive oxygen species (ROS) generation, resulting in a state of oxidative stress, which has been implicated in the development of numerous neurological deficits such as motor and memory dysfunction [7].

It has been proposed that antioxidants protect the brain against tissue impairments induced by CPF intoxication [1]. Beetroot or *Beta vulgaris L.* belongs to the Chenopodiaceae family and is extensively distributed in Europe, in America, and in the Mediterranean countries [8]. Beetroot contains different minerals, vitamins, and phytochemicals such as polyphenols and betalains which are the major active constituents [9]. In folk medicine, different parts of the plant including seeds, leaves, and roots show antioxidant, anti-inflammatory, hepatoprotective, nephroprotective, purgative, carminative, emmenagogue, and wound-healing activities [8]. The antioxidant activity of beetroot can be exploited in the treatment or prevention of CPF-induced neurotoxicity considering the role of ROS generation in brain oxidative challenge and memory deficits. Therefore, the current investigation was conducted to assess the potential beneficial effect of red beetroot methanolic extract (RBR) against CPF-induced neurotoxicity by evaluating the oxidative challenge, the inflammatory status, the apoptotic cascade, and the histological deformations in the cortical tissue of rats.

## 2. Materials and Methods

CPF was supplied by a company for pesticides and chemicals, Riyadh, KSA. CPF was dissolved in Tween20 and then diluted with normal saline (0.9% NaCl); the final gavaged solution contained 5% Tween20. Control rats received the same dose of Tween20 (5% Tween20 in saline). Fresh red beetroot was purchased from a local market in Riyadh, Saudi Arabia, in November 2018. The plant was identified and authenticated by a taxonomy specialist (Botany Department, College of Science, King Saud University, Saudi Arabia). The roots of beetroot (*Beta vulgaris* L.) were washed, dried, and ground using an electrical blender. The fine powder was macerated three times with methanol (70%) for 24 hr at a ratio of 1 : 10 (*w*/*v*). The extract was filtered, and the solvent was removed by vacuum evaporation followed by lyophilization. The RBR was maintained at -80°C until further use.

### 2.1. HPLC Analysis

HPLC (high performance liquid chromatography) analysis was performed using a Waters W600 HPLC system with PDA (Waters 996) detectors to determine the polyphenol and flavonoid content of RBR. The HPLC column was a Luna C18 column (Phenomenex), with a detection wavelength of 280 nm. Elution was carried out using acetic acid (2%; A) and acetonitrile (B). The flow rate was set at 0.8 ml/min throughout the elution. The elution gradient employed was as follows: 0 min, 4% B; 2 min, 6% B; 4 min, 9% B; 6 min, 13% B; 8 min, 15% B; 9 min, 17% B; 10 min, 20% B; 11 min, 21.5% B; 12 min, 24% B; 14 min, 26% B; 16 min, 29% B; 18 min, 100% B; 22 min, 100% B; and 23 min, 7% B. The initial conditions were maintained for 5 min.

### 2.2. Determination of Total Phenolic and Flavonoid Content in Beetroot Extract

The content of total polyphenols (TP) was estimated utilizing the Folin–Ciocalteu method, while the level of total flavonoids (TF) was estimated using the AlCl_3_ method according to the procedures described by Abdel Moneim [10]. Both TP and TF contents were represented as milligram gallic acid equivalents per gram dried weight of the extract (mg GAE/g DW) and milligram quercetin equivalents per gram dried weight of the extract (mg QE/g DW) using the calibration curves of gallic acid and quercetin.

### 2.3. Experimental Design

Male Wistar rats (*n* = 28; 11 week-old; 140–170 g) were placed in polypropylene cages in the animal facility of the Biology Department at PNU (Alriyadh, Saudi Arabia) under controlled conditions (22 ± 2°C and a normal light/dark cycle). All experiments were conducted in accordance with the guidelines of the PN Animal Care Center (H-01-R059/ 190250). Male rats were used in the present study as the male was found to be more sensitive and susceptible to chemical-induced toxicity than the female in the subchronic and chronic studies [11].

The rats were allocated into four groups and gavaged with the respective treatments for 28 consecutive days once daily as follows: group 1 (control group) received physiological saline (0.9% NaCl); group 2 (CPF group) was administered 10 mg/kg CPF solution; group 3 (RBR group) was administered 300 mg/kg RBR dissolved in normal saline which contained 5% Tween20; and group 4 (RBR+CPF group) was administered 300 mg/kg RBR 1 hr before the administration of 10 mg/kg CPF. Rats of all the groups were decapitated swiftly 24 hr after the last dose based on the study conducted by Mahmoud et al. [12]. The brain, liver, kidney, and testis samples were extracted immediately, and the cerebral cortex was dissected, whereas the liver, kidney, and testis samples were stored at -80°C for further investigations. The right cortical hemisphere was homogenized for biochemical investigations, whereas the left hemisphere was prepared for histological analysis.

### 2.4. Biochemical Parameters

#### 2.4.1. Preparation of Tissue Homogenates

A 10% (*w*/*v*) cortical tissue was homogenized in 50 mM Tris-HCl (pH 7.4). The homogenate was centrifuged at 3,000 × *g* for 10 min at 4°C. The obtained supernatant was maintained at -80°C until further use in biochemical experiments. The neuronal protein content was estimated according to the method of Lowry et al. [13] using bovine serum albumin as a standard.

#### 2.4.2. Determination of Acetylcholinesterase (AChE) Activity

The AChE activity was estimated following the protocol described by Ellman et al.'s protocol [14]. The produced thiocholine by the action of acetylcholinesterase on acetyl thiocholine forms 5,5′-dithiobis (2-nitrobenzoic acid) which is the reduced and forms a yellow color thionitrobenzoic acid. The intensity of the developed thionitrobenzoic acid is evaluated spectrophotometrically at 412 nm and is proportional to the activity of AChE in the cortical sample.

#### 2.4.3. Determination of Brain-Derived Neurotrophic Factor (BDNF) and Glial Fibrillary Acidic Protein (GFAP) Levels

The levels of BDNF and GFAP were measured using enzyme-linked immunosorbent assay kits obtained from Cusabio (catalogue number CSB-E04504r) and Merck Millipore (catalogue number NS830) according to the suppliers' protocols, respectively.

#### 2.4.4. Determination of Oxidative Stress Markers

The level of malondialdehyde (MDA), a by-product of lipid peroxidation (LPO), was determined using the thiobarbituric acid method. The thiobarbituric acid reactive substances were estimated at 535 nm and then expressed in terms of MDA produced, as described by Ohkawa and Ohishi [15]. The neuronal level of nitric oxide (NO) was measured by using the Griess reagent (sulfanilic acid and N-(1-naphthyl)ethylenediamine), and the developed azo dye was estimated at 540 nm [16]. The cortical glutathione (GSH) content was assessed using Ellman's reagent, and the absorbance of the developed yellow chromogen was measured at 412 nm [17].

#### 2.4.5. Determination of Antioxidant Enzyme Activities

The activities of cortical glutathione peroxidase (GPx) and glutathione reductase (GR) were estimated according to the methods described by Paglia and Valentine [18] and Factor et al. [19], respectively. The principle of the superoxide dismutase (SOD) assay was based on the ability of SOD to inhibit a nitroblue tetrazolium reduction [20]. Catalase (CAT) activity was measured according to the protocol described by Aebi [21].

#### 2.4.6. Determination of Proinflammation Markers

Neuronal concentrations of interleukin-1*β* (IL-1*β*) and tumor necrosis factor-*α* (TNF-*α*) were estimated using enzyme-linked immunosorbent assay kits purchased from ThermoFisher Scientific (catalogue number ERIL1B) and R&D Systems (catalogue number RTA00). The concentrations of the proinflammatory markers were measured following the manufacturers' procedures.

#### 2.4.7. Determination of Apoptotic Proteins

The concentration of apoptotic proteins including cytochrome c, Bax, and Bcl-2 was measured using enzyme-linked immunosorbent assay kits obtained from Cusabio (catalogue number CSB-EL006328RA), BioVision, Inc. (catalogue number E4513), and Cusabio (catalogue number CSB-E08854r) according to the suppliers' protocols, respectively, while caspase-3 activity is measured by using a calorimetric kit (Sigma-Aldrich, CASP3C-1KT) based on the hydrolysis of Ac-DEVD-pNA by caspase-3, resulting in the release of the p-nitroaniline (pNA) moiety. pNA is detected at 405 nm (*ε*mM = 10.5).

### 2.5. Reverse Transcription-Quantitative Polymerase Chain Reaction (RT-qPCR) Analysis

Total cortical RNA was extracted using the standard TRIzol® procedures (Invitrogen, Carlsbad, CA, USA). RNA was reverse transcribed to cDNA. The primer sequences employed to estimate inducible nitric oxide synthase (iNOS), IL-1*β*, TNF-*α*, Bax, caspase-3, and Bcl-2 gene expressions are listed in Table 1 according to Abdel Moneim [22]. Power SYBR® Green Master Mix was used for RT-qPCR analysis, which was performed in triplicate. The RT-qPCR cycling conditions were 10 min at 95°C followed by 40 cycles involving denaturation at 94°C for 10 s, annealing at 60°C for 30 s, and extension at 72°C for 20 s. Gene expression in the treated groups was presented as a fold change in expression relative to that of the control group. Glyceraldehyde-3-phosphate dehydrogenase was used as the reference gene; its expression remained unaltered throughout the experiment.

### 2.6. Histopathological Examination

The frontal lobe of the cortex was fixed in 10% neutral-buffered formalin for 24 hr at room temperature, dehydrated, paraffinized, sectioned (4–5 *μ*m), and stained with hematoxylin and eosin for light microscopy. Images were captured using a Nikon microscope (Eclipse E200-LED, Tokyo, Japan) at an original magnification of ×400.

### 2.7. Statistical Analysis

The obtained results are expressed as a means ± standard error of mean. Data from different measurements were analyzed using Student's *t*-test and followed by one-way analysis of variance and *post hoc* Duncan's test using a statistical package program (SPSS version 14.0); *P* values < 0.05 indicate statistical significance.

## 3. Results

### 3.1. HPLC Fingerprint of Red Beetroot Extract

**Figure 1:**
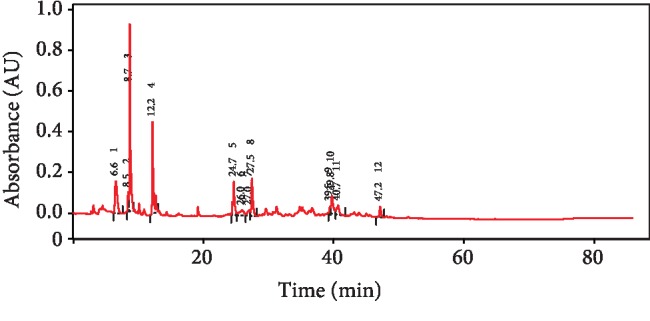
The fingerprint analysis of polyphenol and flavonoid content of red beetroot using HPLC.

The polyphenolic and flavonoid fingerprint of the red beetroot extract determined at 280 nm is showed in Figure 1. The HPLC profile of red beetroot extract shows the presence of 19 peaks with retention times ranging from 0.117 min to 39.457 min. Based on the UV-Visible spectral data and their retention times, the red beetroot extract has a UV band at 280 nm characteristic for polyphenol and flavonoid compounds, possibly cinnamic acid, vanillic, chlorogenic, catechin, caffeic acid, coumaric acid, ferulic acid, rutin, gallic acid, and its derivatives. The observed compounds are consistent with the previous study of Akyol et al. [23].

### 3.2. Total Polyphenolic and Flavonoid Content in Red Beetroot Extract

Table 1 records the total polyphenolic and flavonoid level in RBR. The total polyphenolic content was 91.6 ± 6.36 mg gallic acid equivalent/g fresh beetroot. The total flavonoid level in RBR was 112.3 ± 13.21 mg quercetin equivalents/g fresh beetroot. According to our findings, a nonsignificant variation was observed between the initial and final phenolic and flavonoid levels indicating the stability of RBR throughout the experiment.

### 3.3. Effect of RBR on AChE Activity following CPF Intoxication

**Figure 2:**
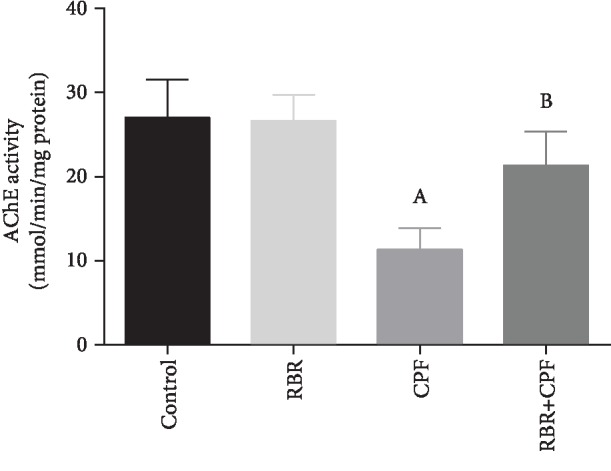
Effect of red beetroot extract (RBR) and/or chlorpyrifos (CPF) on cortical acetylcholinesterase activity (AChE). Data are represented as mean ± SD (*n* = 7); *P* values < 0.05 indicate statistical significance. A; Significant change versus the control group, B; significant change versus the CPF group.

Rats treated with 10 mg/kg CPF for 28 consecutive days showed a significant decrease (*P* < 0.05; *F* = 21.95) in cortical AChE activity compared to that exhibited by rats in the control group. The cholinergic enzyme level did not change significantly in the RBR group. Meanwhile, the rats in the RBR+CPF group exhibited a significantly higher AChE activity than rats of the CPF group; however, the activity was still lower than the normal level (Figure 2).

### 3.4. Effect of RBR on Cortical Brain-Derived Neurotrophic Factor (BDNF) and Glial Fibrillary Acidic Protein (GFAP) following CPF Exposure

**Figure 3:**
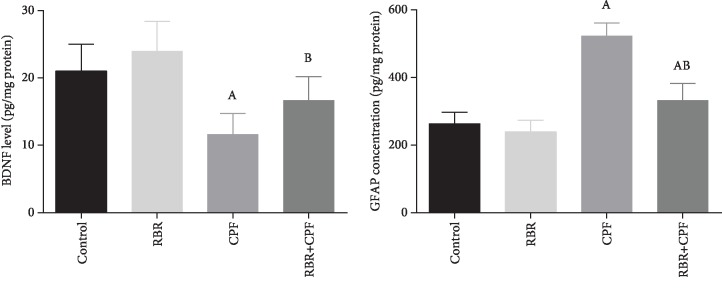
Effect of red beetroot extract (RBR) and/or chlorpyrifos (CPF) on cortical brain-derived neurotrophic factor (BDNF) and glial fibrillary acidic protein (GFAP). Data are represented as mean ± SD (*n* = 7); *P* values < 0.05 indicate statistical significance. A; Significant change versus the control group, B; significant change versus the CPF group.

CPF exposure decreased significantly the level of BDNF in the cortical tissue compared to that in control group (*P* < 0.05). BDNF levels unchanged significantly in the RBR-treated group. Meanwhile, BDNF content was significantly elevated in the RBR+CPF group as compared to those in CPF-exposed animals (Figure 3(a)). GFAP is used as a marker for astroglial activity. ANOVA analysis revealed that rats exposed to CPF showed a significant elevation in the GFAP level in the cortical homogenate compared with that in the control rats (*P* < 0.05). The GFAP level remained unchanged in the RBR-administered group. GFAP was significantly suppressed in the RBR pretreated group compared to CPF-intoxicated rats (Figure 3(b)).

### 3.5. Effect of RBR on CPF-Induced Oxidative Stress in the Brain Tissue

**Figure 4:**
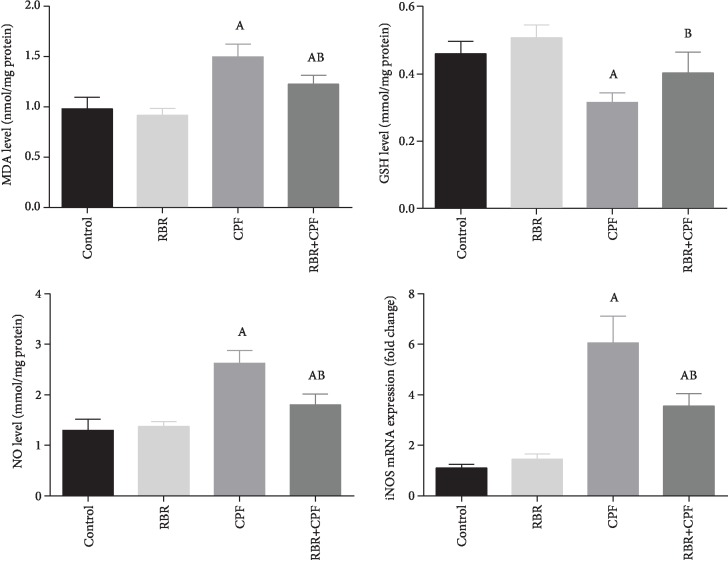
The protective effect of red beetroot extract (RBR) on the cortical lipid peroxidation (MDA), glutathione (GSH) content, nitric oxide (NO), and inducible nitric oxide synthase (iNOS) expression in response to chlorpyrifos (CPF) exposition. Data are expressed as mean ± SD (*n* = 7); *P* values < 0.05 indicate statistical significance. A; Significant change versus the control group, B; significant change versus the CPF-injected group. Results of iNOS mRNA expression are expressed as mean ± SD of triplicate assays and were normalized to GAPDH and expressed as fold change (log2 scale), with respect to mRNA levels in the control and the CPF groups.

**Figure 5:**
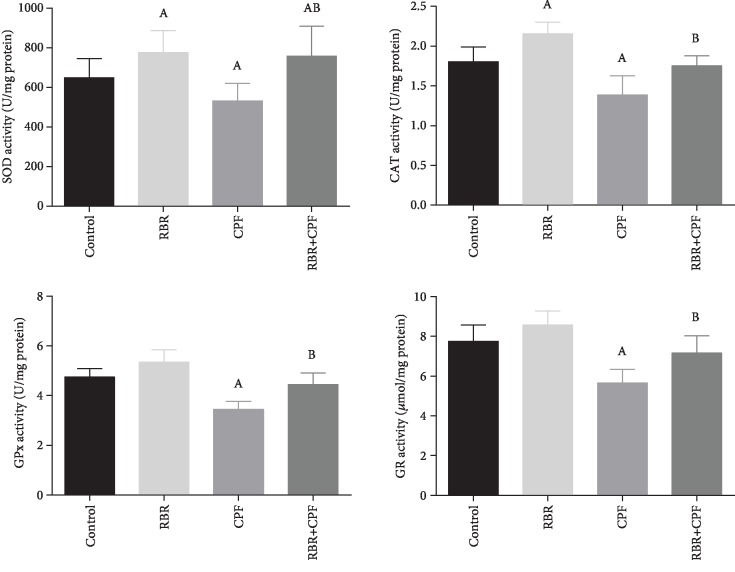
The protective effect of red beetroot extract (RBR) on the cortical glutathione peroxidase (GPx), glutathione reductase (GR), superoxide dismutase (SOD), and catalase (CAT) activities following chlorpyrifos (CPF) intoxication. Data are expressed as mean ± SD (*n* = 7); *P* values < 0.05 indicate statistical significance. A; Significant change versus the control group, B; significant change versus the CPF group.

To investigate the potential antioxidant capacity of RBR against CPF-induced oxidative reaction in cortical tissues, the prooxidants (malondialdehyde (MDA), the by-product of lipid peroxidation; inducible nitric oxide synthase (iNOS) mRNA expression, and nitric oxide (NO)) and antioxidants (glutathione (GSH), superoxide dismutase (SOD), catalase (CAT), glutathione peroxidase (GPx), and glutathione reductase (GR)) were assessed. In the CPF group, CPF potentiated oxidative challenge in the neuronal tissue as demonstrated by the significant elevation in MDA, iNOS, and NO content (*P* < 0.05; *F* = 27.06, 87.03, and 35.83, respectively) accompanied by a decrease in the GSH content and activities of SOD, CAT, GPx, and GR (*P* < 0.05; *F* = 14.8, 3.84, 12.2, 15.27, and 10.1, respectively) relative to the values of the control. Rats treated with RBR showed a nonsignificant change in the oxidants but increased SOD and CAT activities. The pretreatment with 300 mg/kg RBR for 28 consecutive days resulted in a marked decrease in the levels of prooxidants in the brain tissue and a significant increase (*P* < 0.05) in the levels of enzymatic and nonenzymatic antioxidant molecules, relative to those of the CPF-intoxicated rats (Figures 4 and 5).

### 3.6. Effect of RBR on Inflammatory Response following CPF Exposure

**Figure 6:**
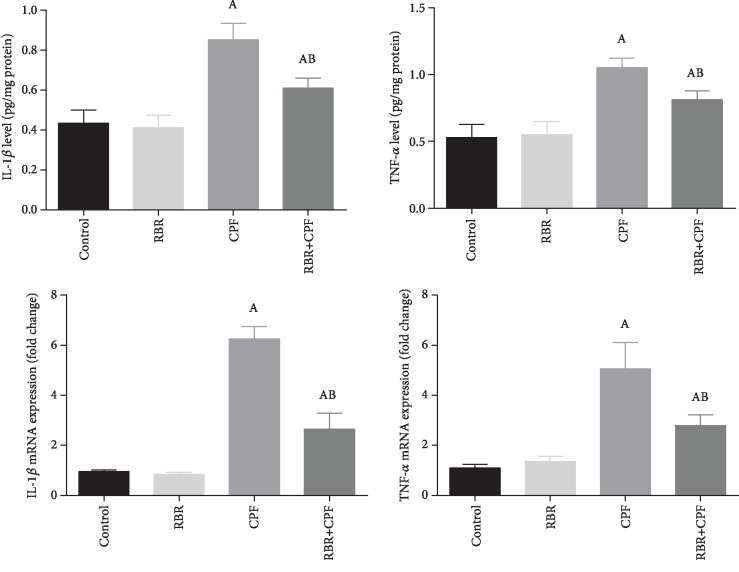
The protective effect of red beetroot extract (RBR) on the cortical interleukin-1*β* (IL-1*β*) and tumor necrosis factor-*α* (TNF-*α*) in response to chlorpyrifos (CPF) intoxication. Data are expressed as mean ± SD (*n* = 7); *P* values < 0.05 indicate statistical significance. A; Significant change versus the control group, B; significant change versus the CPF-injected group. Results of mRNA expression are expressed as mean ± SD of triplicate assays and were normalized to GAPDH and expressed as fold change (log2 scale), with respect to mRNA levels in the control and the CPF groups.

The concentration of IL-1*β* and TNF-*α* was estimated in the cortical homogenate to assess the neuroinflammation status in rats treated with RBR before CPF administration. The treatment with CPF significantly (*P* < 0.05; *F* = 32.22 and 34.54, respectively) increased the levels of the assessed proinflammatory cytokines in comparison with the levels of the control group. RBR-treated rats showed a nonsignificant alteration in these mediators unlike rats in the control group. The treatment with RBR prior to the administration of CPF resulted in significantly lower levels of the chemical mediators than those of the CPF-intoxicated rats. To further explore the molecular anti-inflammatory activity of RBR against CPF intoxication, IL-1*β* and TNF-*α* gene expression in the cortical tissue was evaluated using qRT-PCR analysis. CPF exposure led to the mRNA overexpression of both cytokines significantly (*P* < 0.05; *F* = 231.36 and 57.53, respectively). Meanwhile, rats in the RBR+CPF group showed significantly downregulated expression of IL-1*β* and TNF-*α*, demonstrating the anti-inflammatory activity of RBR against the inflammatory response produced by CPF (Figure 6).

### 3.7. Effect of RBR on Apoptotic Protein Expression in the Brain Tissue following CPF Exposure

**Figure 7:**
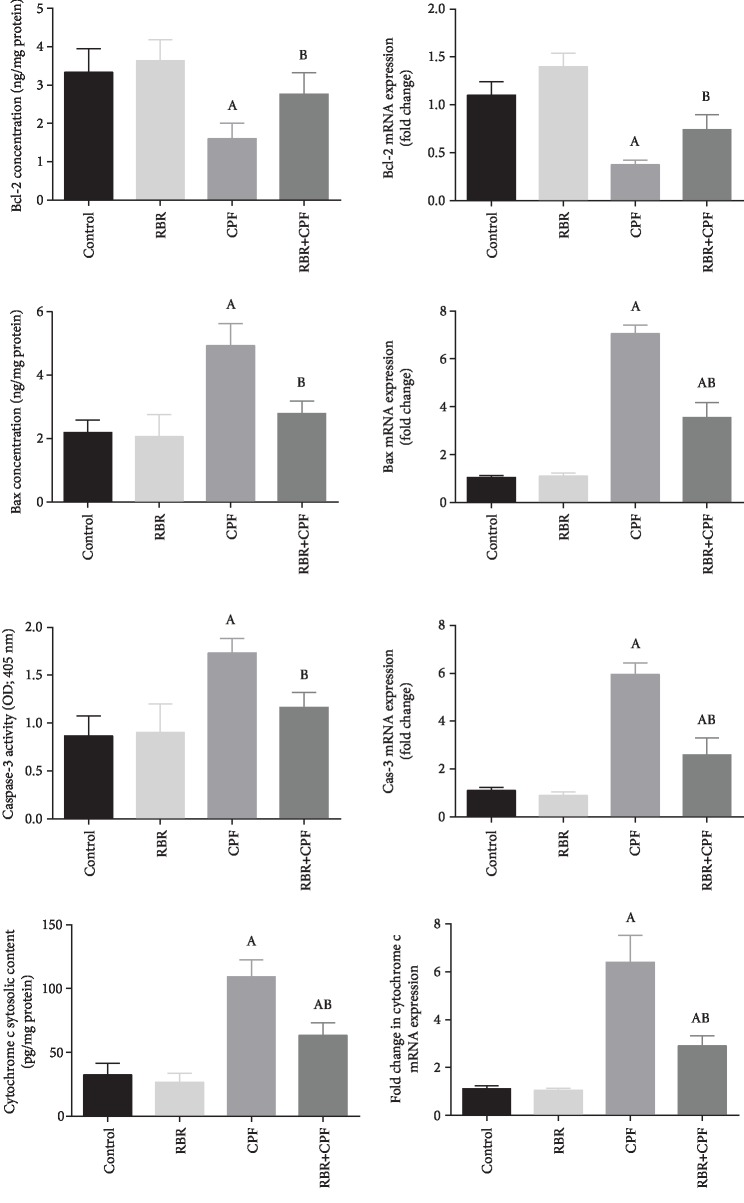
The protective effect of red beetroot extract (RBR) on the cortical protein and gene expression of cytochrome c, Bax, caspase-3, and Bcl-2 in response to chlorpyrifos (CPF) exposition. Data of protein expression are expressed as the mean ± SEM (*n* = 7), whereas mRNA expression data are expressed as the mean ± SEM of triplicate assays, normalized to the GAPDH, and expressed as fold change (log2 scale), with respect to mRNA levels in the control group, *P* values < 0.05 indicate statistical significance. A; Significant change versus the control group, B; significant change versus the CPF group.

**Figure 8:**
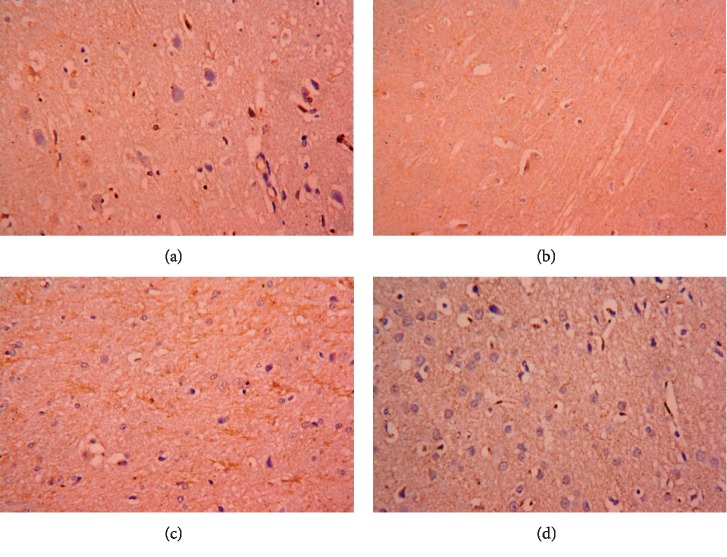
Cortical expression of caspase-3 following the treatment with red beetroot extract (RBR) and/or chlorpyrifos (CPF) was evaluated using immunohistochemical staining. (a) represents the control group; (b) represents the RBR-treated group; (c) represents the CPF-treated group; (d) represents the RBR+CPF-5treated group.

qRT-PCR and ELISA analysis revealed that, CPF treatment increased significantly the level of cytochrome c, upregulated the expression of proapoptotic proteins including Bax and caspase-3 and downregulated that of Bcl-2, the antiapoptotic protein (*P* < 0.05; *F* = 345.61, 166.69, and 70.95, respectively). RBR administration did not change the cytochrome c level, expression of the pro- and antiapoptotic proteins in the brain tissue. Meanwhile, levels of cytochrome c, Bax, and caspase-3 were downregulated and Bcl-2 was upregulated in the RBR+CPF group compared to the levels in the CPF group (Figure 6). Moreover, the expression of caspase-3 was estimated in the cortical tissue using an immunohistochemistry method. CPF was found to upregulate this proapoptotic protein as compared to the control. Meanwhile, it has been downregulated in the RBR+CPF-treated group as compared to CPF-intoxicated group (Figures 7 and 8).

### 3.8. Histopathological Alterations in the Brain Tissue following CPF Exposure

**Figure 9:**
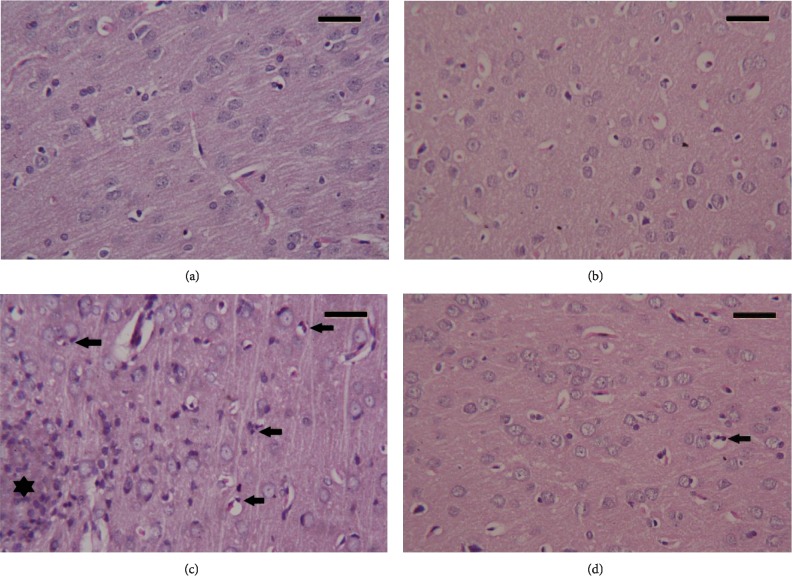
Histopathological observations in cortical tissue following the treatment with red beetroot extract (RBR) and chlorpyrifos (CPF). (a) Photomicrograph of the cortical tissue of the control group showing healthy cortical structure. (b) Photomicrograph of the cortical tissue of rats treated with RBR alone showing a healthy histological structure. (c) Photomicrograph of the cortical tissue of rats exposed to CPF showing degenerative alterations in neurons, and a large number of eosinophil leukocyte infiltrating (black star) between neurons and apoptotic cells (arrow) have been observed. (d) Photomicrograph of the cortical tissue of rats treated with RBR and CPF showing a recovery of cortical tissue; however, some neurons are showing a degree of degeneration with infiltrating eosinophil. Sections were stained with hematoxylin and eosin (400x). Scale bar = 50.

Cortical tissue of the control group and the RBR-treated group showed a normal characteristic structure. CPF-intoxicated animals exhibited noticeable pyknotic, degenerated, and shrunken neurons associated with the presence of some apoptotic neurons. Serious inflammatory cell infiltration was also observed in the CPF group. Meanwhile, pretreatment with RBR improved the cortical deformations induced by CPF; however, some neurons remained damaged (Figure 9).

## 4. Discussion

Organophosphorus compounds are used for various military, household, and agricultural purposes to control and prevent different disease vectors such as flies, cockroaches, beetles, helminthes, cockroaches, termites, and corn rootworms [24]. Exposure to organophosphorus is strongly associated with the loss of cellular functions and the development of different diseases such as cardiovascular, renal, respiratory, and neurodegenerative disorders [25]. CPF is a wide-spectrum organophosphorus insecticide, and its indoor and outdoor uses cause several side effects to animals and humans [24]. Our investigation was designed to study the neurotoxic profile of CPF and the potential neuroprotective effect of RBR in rats. In the current study, CPF disturbed the cholinergic transmission by suppressing the activity of AChE. In addition, CPF also decreased the BDNF and increased GFAP cortical levels. AChE plays an essential role in acetylcholine metabolism, and its dysregulation causes different pathological conditions. CPF at different doses can inhibit AChE in different experimental models, leading to the accumulation of acetylcholine at synaptosomes and thus producing memory and learning impairments [7, 25]. It has been demonstrated that CPF is able to inhibit AChE activity by enhancing the phosphorylation of its serine site [26].

BDNF plays a key role in the survival of nerve cells and synaptic plasticity and has been involved in several neurological impairments such as Huntington, Alzheimer, and epilepsy [27]. CPF has been reported to inhibit the expression of BDNF in the cortical tissue of young rats [28]. This effect has been attributed to the downregulation of BDNF mRNA expression [29]. GFAP represents the main filament in the astrocytic cytoskeleton and has been used widely as a marker for neuroinflammation and astrocyte activation [30]. It has been reported that CPF activates microglia and the overproduction of the proinflammatory cytokines which activates astrocytes and upregulates GFAP mRNA expression in different brain regions [31]. Interestingly, RBR treatment significantly modulated AChE activity against CPF-induced cholinergic dysfunction in this study. Hajihosseini et al. [32] demonstrated that beetroot leaf extract improved neuronal damage and memory dysfunction induced by scopolamine injection, through the enhancement of cholinergic activity in the brain tissue. Moreover, *B. vulgaris* leaf extract mediated cholinergic neurotransmission in rats with impaired memory [33]. In addition, *B. vulgaris* modulated significantly AChE activity in an *in vitro* study [34]. It has been reported that flavonoids target astrocytes and enhance the secretion of BDNF and inhibit the expression of GFAP in the central nervous system [35]. This may explain the ability of RBR to increase BDNF and decrease GFAP levels following CPF exposure in the current study.

Of all the body organs, the brain has the closest association with the progression of oxidative stress conditions due to its high lipid content, low antioxidant molecule level, and high oxygen consumption [36]. Exposure to environmental toxicants causes neuronal damage and behavioral deficits [37]. In the present study, a disturbance in the redox homeostasis was observed in the cortical tissue following subchronic CPF exposure, as demonstrated by the elevated levels of MDA and NO. MDA is the by-product of lipid peroxidation and used as a marker for oxidative stress. NO is a biological mediator and has a fundamental role in different physiological functions and serves as nitrosative stress.

GSH is the major nonenzymatic antioxidant molecule that interacts and neutralizes the oxidizing compounds, and its reduction has been linked with the progression of oxidative stress [38, 39]. SOD represents the first preventive antioxidant enzyme that neutralizes singlet oxygen and spontaneously dismutates superoxide radicals to hydrogen peroxide. The decomposition of hydrogen peroxide is successfully accomplished by CAT and hence prevents lipid peroxidation [40]. GPx together with GSH catalyzes the reduction of hydrogen peroxide and lipid peroxides, whereas GR promotes the NADPH-driven conversion of GSSG to GSH [41]. A depletion of these antioxidant enzymes and molecules could be associated with an overwhelming accumulation of hydrogen peroxide that suppresses neuronal antioxidant defense systems [12]. The increase in the levels of the assessed prooxidants was accompanied by a decrease in the GSH content. CPF reduced the activities of the enzymatic antioxidant defense molecules, namely, GPx, SOD, and GR.

Previous reports verified the involvement of oxidative stress in CPF-induced neuronal damage [42, 43]. CPF enhances the overproduction of ROS and consequently triggers polyunsaturated lipid5 peroxidation in the brain and other tissues [12]. The elevation in the production of NO has been observed in the brain tissue in response to CPF treatment [25]. This increase has been attributed to the overexpression of iNOS which potentiates the synthesis of NO in the brain tissue [44]. A decrease in GSH content and inhibition of GPx, GR, SOD, and CAT activities have been recorded in different models following CPF intoxication [7, 45]. The antioxidant enzymes protect the cell against different free radicals generated, following the exposure to chemicals and environmental toxicants. Several reports showed that, upon exposure to organophosphorus compounds, the functions of the antioxidant molecules are impaired, and hence, they fail to prevent the development of oxidative stress in different brain regions [7, 42, 43]. The inhibition of these antioxidant enzymes has been linked with an increase in lipid peroxidation [46].

Due to its antioxidant content, particularly betalain, [47], RBR decreased LPO along with NO levels while it increased the GSH content in the brain tissue of rats in the RBR+CPF group. This was accompanied by the enhanced activities of GPx, GR, SOD, and CAT. Beetroot has antioxidant properties against different types of free radicals [48]. Beetroot leave extract decreased the MDA level and enhanced total antioxidant capacity in rats treated with scopolamine [32]. In another study, beetroot extract decreased the MDA level and increased the activity of catalase in gentamicin-induced oxidative stress in renal tissue [49]. The consumption of beetroot has been associated with the prevention of oxidative reactions following xenobiotics' exposure in different models, through the inhibition of prooxidants and the activation of the endogenous antioxidant system [47]. Furthermore, betalain suppressed the peroxidation of membrane lipids enhanced by free iron and H_2_O_2_-activated metmyoglobin [50]. In addition, betanin inhibited iNOS expression in renal tissue intoxicated with paraquat [51]. The increase in the levels of antioxidant enzymes following beetroot administration is due to its ability to activate nuclear factor (erythroid-derived 2)-like 2 which further activates the gene expression of antioxidant enzymes [52].

Neuroinflammation has been observed following CPF intoxication. In the present study, CPF activated an immune response in the cortical tissue as indicated by the increase in the levels of proinflammatory cytokines—IL-1*β* and TNF-*α*. Interleukin-1*β* is a proinflammatory cytokine released in the brain from astrocytes, microglia, and Schwann cells. It plays fundamental roles in acute and chronic inflammatory, pain, and autoimmune disorders [53]. TNF-*α* is also a proinflammatory cytokine produced mainly from microglia and astrocytes. It regulates different physiological functions in the central nervous system including synaptic plasticity, memory, and learning [27]. Oversecretion of IL-1*β* and TNF-*α* is associated with several pathological conditions [54].

Exposure to organophosphorus pesticides is associated with excessive release of IL-1*β* and TNF-*α* through the activation of glial cells [55]. Organophosphorus compounds upregulated the expression of genes involved in cytokine signaling [56]. Tian et al. [57] showed that CPF enhanced the secretion of the proinflammatory mediators through the activation of nuclear factor kappa B (NF-*κ*B) in the brain tissue of neonatal rats. The anti-inflammatory agents have the potential to provide protection against organophosphorus insecticide-induced neurotoxicity [58]. It has been demonstrated that beetroot extracts and the active betalain compounds possess anti-inflammatory activity mediated by the signaling of the proinflammatory cytokines [47]. Indicaxanthin which belongs to the group of betalains found in beetroot inhibited the carrageenin-induced inflammatory response by reducing the levels of IL-1*β* and TNF-*α* and by deactivating the NF-*κ*B pathway [59]. Moreover, treatment with beetroot inhibited TNF-*α* and IL-6 in renal tissue exposed to gentamicin through the inactivation of NF-*κ*B [49, 60].

The involvement of the apoptotic pathway was demonstrated in the current study, CPF intoxication upregulated the expression of Bax and caspase-3 while the expression of Bcl-2 was downregulated in the cortical tissue. Mohamed et al. [61] reported that chronic CPF exposure enhanced the expression of proapoptotic proteins including Bax and caspase-3 in hepatic tissues of rats. In another model, CPF potentiated the overexpression of Bax and caspase-3 in human neural precursor cells, while the expression of Bcl-2 was downregulated. This apoptotic action has been attributed to ROS overproduction and the activation of NF-*κ*B [62]. It has been reported that the generation of ROS induced by CPF triggers the upregulation of proapoptotic proteins like Bax and caspase-3, and the downregulation of antiapoptotic proteins like Bcl-2 [63]. In the current study, RBR inhibited the expression of Bax and caspase-3 and enhanced the expression of Bcl-2 in the cortical tissue. Similarly, El Gamal et al. [49] reported the upregulation and the downregulation of the antiapoptotic protein and the proapoptotic protein expressions, respectively, in response to gentamicin in the renal tissue. We suggest that the antiapoptotic effect of RBR might be due to its ability to scavenge ROS induced by CPF intoxication.

## 5. Conclusion

In conclusion, the current study suggests that RBR is a potential neuroprotective agent in CPF-induced cortical damage mediated by the regulation of AChE activity, increasing BDNF, decreasing GFAP, inhibition of prooxidants (LPO, iNOS, and NO), and enhancement of the cortical endogenous antioxidant molecules (GSH, GPx, GR, SOD, and CAT). The protective effect of RBR also involved the suppression of the expression and levels of IL-1*β* and TNF-*α*, enhancement of the expression of Bcl-2, and downregulation of the expression of Bax and caspase-3. These results substantiate the use of red beetroot extract against CPF-induced neurotoxicity in rats.

## Figures and Tables

**Table 1:** Primer sequences of genes analyzed in real time PCR.

Name	Accession number	Sense (5′---3′)	Antisense (5′---3′)
GAPDH	NM_017008.4	GCATCTTCTTGTGCAGTGCC	GATGGTGATGGGTTTCCCGT
iNOS	NM_012611.3	GTTCCTCAGGCTTGGGTCTT	TGGGGGAACACAGTAATGGC
IL-1*β*	NM_031512.2	GACTTCACCATGGAACCCGT	GGAGACTGCCCATTCTCGAC
TNF-*α*	NM_012675.3	GGCTTTCGGAACTCACTGGA	CCCGTAGGGCGATTACAGTC
Bcl-2	NM_016993.1	ACTCTTCAGGGATGGGGTGA	TGACATCTCCCTGTTGACGC
Bax	NM_017059.2	GGGCCTTTTTGCTACAGGGT	TTCTTGGTGGATGCGTCCTG
Caspase-3	NM_012922.2	GAGCTTGGAACGCGAAGAAA	TAACCGGGTGCGGTAGAGTA
Cytochrome c	NM_012839.2	CTTGGGCTAGAGAGCGGGA	TGAAGCACGGGTGAGTCTTC

The abbreviations of the genes; GAPDH: glyceraldehyde-3-phosphate dehydrogenase; iNOS: inducible nitric oxide synthase; IL-1*β*: interleukin 1 beta; TNF-*α*: tumor necrosis factor-*α*; Bcl-2: b-cell lymphoma 2; Bax: Bcl-2-like protein 4.

## Data Availability

All relevant data are within the paper.
